# Changes in vocal parameters with social context in humpback whales: considering the effect of bystanders

**DOI:** 10.1007/s00265-016-2108-0

**Published:** 2016-04-02

**Authors:** Rebecca A. Dunlop

**Affiliations:** Cetacean Ecology and Acoustics Laboratory, School of Veterinary Science, The University of Queensland, Gatton, Queensland 4343 Australia

**Keywords:** Frequency coding, Source level, Social group, Signal design, Vocal communication

## Abstract

**Abstract:**

Many theories and communication models developed from terrestrial studies focus on a simple dyadic exchange between a sender and receiver. During social interactions, the “frequency code” hypothesis suggests that frequency characteristics of vocal signals can simultaneously encode for static signaler attributes (size or sex) and dynamic information, such as motivation or emotional state. However, the additional presence of a bystander may result in a change of signaling behavior if the costs and benefits associated with the presence of this bystander are different from that of a simple dyad. In this study, two common humpback whale social calls (“wops” and “grumbles”) were tested for differences related to group social behavior and the presence of bystanders. “Wop” parameters were stable with group social behavior, but were emitted at lower (14 dB) levels in the presence of a nearby singing whale compared to when a singing whale was not in the area. “Grumbles” were emitted at lower (30–39 Hz) fundamental frequencies in affiliative compared to non-affiliative groups and, in the presence of a nearby singing whale, were also emitted at lower (14 dB) levels. Vocal rates did not significantly change. The results suggest that, in humpbacks, the frequency in certain sound types relates to the social behavior of the vocalizing group, implying a frequency code system. The presence of a nearby audible bystander (a singing whale) had no effect on this frequency code, but by reducing their acoustic level, the signal-to-noise ratio at the singer would have been below 0, making it difficult for the singer to audibly detect the group.

**Significance statement:**

The frequency, duration, and amplitude parameters of humpback whale social vocalizations were tested between different social contexts: group social behavior (affiliating versus non-affiliating), the presence of a nearby singing whale, and the presence of a nearby non-singing group. “Grumbles” (commonly heard low-frequency unmodulated sounds) frequencies were lower in affiliating groups compared to non-affiliating groups, suggesting a change in group motivation (such as levels of aggression). “Wop” (another common sound type) structure (frequency and duration) was similar in affiliating and non-affiliating groups. In the presence of an audible bystander (a singing whale), both sound types were emitted at similar rates, but much lower amplitudes (14 dB), vastly reducing the detectability of these sounds by the singer. This suggests that these groups were acoustically avoiding the singing whale. They did not, however, acoustically respond to the presence of a nearby non-singing group.

## Introduction

Historically, theories and models developed from studies in animal communication were based on a dyad of one sender and one receiver (McGregor and Dabelsteen 1996). The underlying assumption in these studies was that communication was defined by the transmission of information by one individual (the sender) to another individual (the receiver) who uses the information to influence its decisions or behavior (Bradbury and Vehrencamp 1998). The “frequency code hypothesis” suggests that certain features of a vocal signal can encode static information on caller attributes such as size while simultaneously encoding dynamic information such as motivation (Ohala 1984; for extensive review, see Taylor and Reby 2010). Static signaler information, such as size or sex, should therefore be reflected in static signal components (Fitch 1997; Reby et al. 1999; Reide and Fitch 1999; Reby and McComb 2003; Harris et al. 2006; Lemasson et al. 2009). Dynamic motivational information, relating to the social context of the signaler, can also be encoded in certain flexible structural features of the sound (Poole et al. 1988; Fischer et al. 2002; Notman and Rendell 2005; Suigiura 2007; Soltis et al. 2011). This static–dynamic coding system has been shown to exist in various taxa. By-product “static” components, such as formants, can indicate the signaler’s body size, weight, or dominance rank (e.g., male fallow deer, *Dama dama*: Vannoni and McElligott 2008; Charlton and Reby 2011; baboons, *Papio cynocephalus ursinus*: Fischer et al. 2004; and domestic dogs, *Canis familiaris*: Taylor et al. 2011). Dynamic features of calls have been found to encode motivational cues in species such as the domestic piglet, *Sus scrofa domestica* (Linhart et al. 2015); the African elephant, *Loxodonta Africana* (McComb et al. 2003; Soltis et al. 2009); and spotted hyenas, *Crocuta crocuta* (Theis et al. 2007). Levels of arousal, for example, may be encoded in parameters such as call rate (e.g., red deer: Clutton-Brock and Albon 1979), amplitude (e.g., the domestic piglet, *S. scrofa domestica*: Linhart et al. 2015; the African elephant, *L. Africana*: Soltis et al. 2011), and frequency (e.g., the domestic horses, *Equus caballus*, Briefer et al. 2015; Geoffroy’s spider monkeys, *Ateles geoffroyi*: Ordonez-Gomez et al. 2015).

Many previous studies have shown that vocal communication is not a simple dyadic exchange between a sender and receiver, but is a communication network involving a potential “audience” (for an extensive review, see Fichtel and Manser 2010). This “audience” may or may not be the intended recipients of the signal. There are numerous examples of this in various taxa ranging from Siamese fighting fish (*Betta spendens*), who modify their dyadic display behavior depending on the sex of the audience (Doutrelant et al. 2001; Dzieweczynski et al. 2005; Dzieweczynski and Walsh 2011), to chimpanzees (*Pan troglodytes*; Townsend and Zuberbühler 2009; Fedurek et al. 2013), which are thought to understand third-party relationships, or “triadic awareness” (Slocombe and Zuberbühler 2007). “Private” signals are defined as being quiet signals that attenuate over distance (Dabelsteen 2005) and therefore usually used for close-range communication between conspecifics. Social calls that are used to mediate group affiliations (Cheney et al. 1995; Fedurek et al. 2013) and promote group cohesion (Palombit 1992; Holland et al. 1998; Dabelsteen 2005; Riesch et al. 2008) fall into this category of vocal signaling. Although vocal signals between a sender and receiver may be assumed to be “private,” within the signal range, information from the signal may be available to other individuals not directly involved in the group. The presence of these other receivers (or “bystanders”) may result in a change of signaling behavior if the costs and benefits associated with the presence of an audience are different from that within a simple dyad (Marler et al. 1986; McGregor and Peake 2000). These bystanders could be considered to be “eavesdroppers” if unintended information is passed on (McGregor et al. 1999; Dabelsteen 2005; Peake 2005). Studies in black-capped chickadees (*Poecile atricapillus*), for example, found that males tended to eavesdrop on vocal interactions outside their own territories (Fitzsimmons et al. 2008) and then used the information gained to modify their own behavior (Vignal et al. 2004).

Humpback whales are a particularly vocal large baleen whale species in that they “sing” (Payne and McVay 1971) as well as produce a large number of different non-song vocal sounds (Dunlop et al. 2007). Humpback whale song is a male-only signal (Darling et al. 1983; Glockner 1983; Baker 1994; Darling and Bérubé 2001), defined as being long, complex, repetitive, and highly stereotyped (Payne et al. 1983; Cato 1991) and produced at a high acoustic level, therefore audible over tens of kilometers (Au et al. 2006). Non-song “social vocalizations” in humpback whales are not clearly structured like song as they have no serial patterning and are heard as single sounds or in short bursts (Tyack 1983; Tyack and Whitehead 1983; Silber 1986; Dunlop et al. 2007; Rekdahl et al. 2015). Humpback whales utilize an extremely variable catalogue of social vocalizations, from almost infra-sonic “grumbles” to high-frequency “chirp”-like sounds (Dunlop et al. 2007), and these sounds are used by both sexes and in closer-range communication compared to song (Dunlop et al. 2008). Although earlier work assumed that these sounds were produced only in aggressive and/or competitive social encounters (Tyack 1983; Tyack and Whitehead 1983; Baker and Herman 1984; Silber 1986), later studies found that they are used in various other social and behavioral contexts, such as between a female and her calf or from single animals that were not part of a group (Dunlop et al. 2008).

During migration, humpback whale social interactions are characterized by frequent changes in group membership (affiliations). These social affiliations, to some extent, are mediated by “social sounds” (Tyack 1983; Tyack and Whitehead 1983; Baker and Herman 1984; Silber 1986; Dunlop et al. 2008). Common social groups during migration and on breeding grounds include lone singing males, “competitive” groups (where a number of males are thought to be competing for access to one female), female–calf pairs with or without a male “escort” (Tyack and Whitehead 1983; Baker and Herman 1984), and adult pairs, some of which are thought to be males “consorting” with non-lactating females (Herman and Antinoja 1977; Darling et al. 1983; Glockner 1983; Clapham et al. 1992; Clapham 1996). Singing whales are sometimes joined by other males (in which case they usually stop singing), or can stop singing and then join other groups (Darling and Bérubé 2001). During the southward migration of the East Australian humpback whale population, instances of singing males joining and continuing to sing when escorting female–calf pairs (“singing escorts”) are often seen (Noad 2002; Smith et al. 2008). This fluid social system provides the opportunity to look for a context-driven dynamic component in vocal coding within a large marine mammal species during social affiliations. Given the variety in humpback whale social group structure, it is likely that attributes of commonly heard non-song vocalizations (such as vocal rate, parameters of the fundamental frequency, and/or the level at which they are produced) change according to the social behavior of the vocalizing group. While these affiliations are taking place, other groups and singing whales are present in the area, forming a potential communication network. Male singing whales (those not involved in the affiliation), if close to the group, could be considered to be “bystanders” as they would be audible to the group, but not involved in the affiliation. Other non-singing groups in the area may or may not be audible to the group depending on how far away they are and whether or not they are emitting audible sounds.

Using observational data, the effect of social behavior on vocal rate, frequency, duration, and level was determined by comparing vocalizations from non-affiliating groups to affiliating groups, taking into account the group social structure. In addition, the presence of nearby singing whales (which would have been audible to the vocalizing group) and other nearby whales or groups of whales was considered to determine whether the presence of bystanders changed this group’s vocal behavior.

## Methodology

### Visual and acoustic data collection

The eastern Australian population of humpback whales migrates annually along the eastern Australian coastline between feeding areas in the Antarctic and breeding grounds inside the Great Barrier Reef off central Queensland. During their southward migration in particular, they pass close to shore in the vicinity of Peregian Beach, 130 km north of Brisbane on the east coast, where this study was conducted. Data were collected as part of the Humpback Whale Acoustic Research Collaboration (HARC) project during the September/October southward migrations in 2002, 2003, 2004, 2008, and 2009. Additional data were collected as part of the BRAHSS study (Behavioural Response of Australian Humpback Whales to Seismic Surveys) in 2010 and 2011 from the same location.

Land-based observations (including the position, composition, and behaviors) of all migrating groups passing through the study area were collected daily (7 am to 5 pm, weather permitting) from an elevated survey point (73-m elevation). A theodolite (Leica TM 1100) was used in conjunction with a notebook computer running *Cyclopes* software (E. Kniest, Univ. Newcastle, Australia) to track the groups in real time. Each theodolite fix was time-stamped and the behavior of the fixed whale (e.g., blow, breach, pectoral slap, tail slap, etc.), group composition, direction of travel, and any other notes of interest (e.g., splitting or joining of groups) were recorded with each fix. Observers, using binoculars, made additional notes. These observations were also recorded onto *Cyclopes* in real time. Weather was noted hourly and observations included sea state, wind speed and direction, cloud cover, glare strength and position, swell height and direction, and rainfall.

Acoustic recordings were made from three to five hydrophone buoys moored in 18–28 m of water and arranged in a line or T-shaped array. Each hydrophone buoy consisted of a surface buoy containing a custom-built pre-amplifier (+20-dB gain) and 41B sonobuoy VHF radio transmitter. A High Tech HTI-96-MIN hydrophone with built-in +40-dB pre-amplifier was suspended approximately 1 m above each buoy’s mooring and its cable ran up the anchor rope to the buoy where it was connected to the pre-amplifier and transmitter. Buoys 1–3 were approximately 750 m apart and were arranged in a line parallel to, and 1.5 km from, the beach. Buoys 4 and 5 were moored seaward from buoy 2 approximately 600 m apart, forming a line perpendicular to that of buoys 1–3. Signals were received onshore at a base station using a directional antenna and type 8101 four-channel sonobuoy receiver. This was connected to a PC; acoustic data were recorded to hard disk via a series E National Instruments Data Acquisition Card and recorded using *Ishmael* acoustic tracking software (D. Mellinger, Oregon State Univ.) usually at a sampling rate of 22 kHz.

*Ishmael* was also used to determine the location of sound sources detected. This was achieved by cross-correlation of the same sound arriving at the different hydrophones to determine differences in the arrival time of the sound at the buoys. These differences, together with an accurate knowledge of the positions of the hydrophones (surveyed accurately at the start of each season using cross bearings from two theodolites at known points on the beach), were then used to determine the most likely location of the source (e.g., singing or vocalizing whales). Small errors in determining the time of arrival differences can result in errors in the distance measurements to the source (although the bearing is usually robust). However, sound location accuracy was significantly improved by taking the mean position of several estimates over a brief period and by using more than three buoys (Noad et al. 2004).

Land-based and base station computers were linked in real time using a wireless network. Usually, no more than six groups were migrating through the study area at any one time and, unless interacting with each other (affiliating), were usually more than 1.5 km from each other. Therefore, theodolite tracking from the land-based station paired with acoustic tracking from the base station provided adequate accuracy of position fixing to ensure that there was no ambiguity as to which visually tracked group was vocalizing.

### Measurement of social vocalizations

**Fig. 1:**
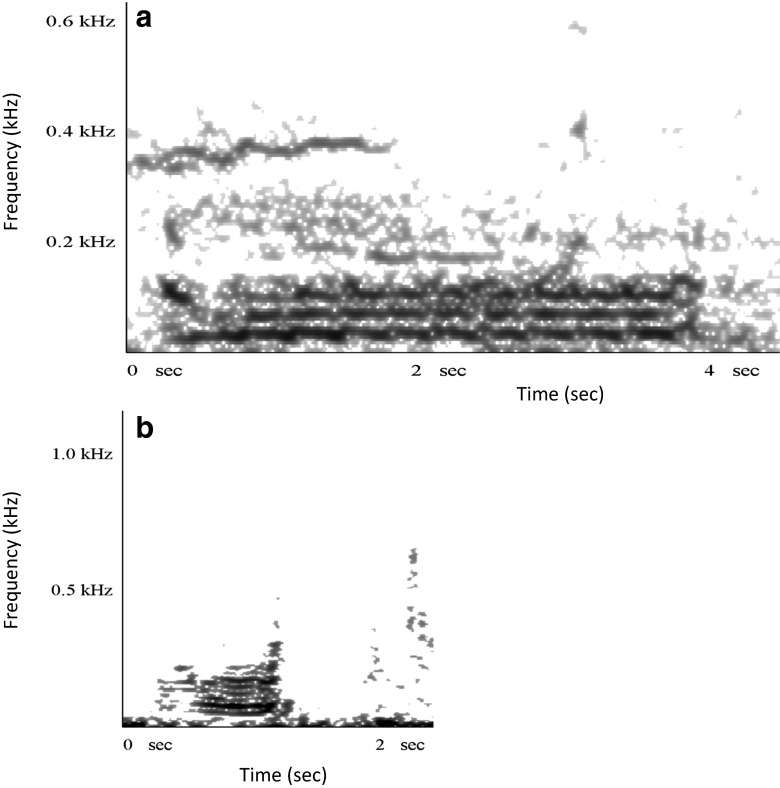
Spectrograms (4096 point FFT yielding 5.4-Hz frequency resolution) of a “grumble” (**a**) and “wop” (**b**) vocal sound

Non-song vocalizations were isolated from acoustic array recordings when they were tracked acoustically to specific groups. Vocalizations were initially subjectively classified into different types based on aural and spectrographic characteristics and then statistically classified using discriminant function analysis (Dunlop et al. 2007) and again using a classification tree (Rekdahl et al. 2013). “Grumbles” are low-frequency (fundamentally generally below 80 Hz) sounds with little or no frequency modulation lasting more than 0.5 s (Fig. 1). “Wops” are audibly distinct, short, low-frequency (fundamentally *<*60 Hz) upsweeps (Dunlop et al. 2007) and are the predominant call in groups containing a female and a calf (Dunlop et al. 2008; Fig. 1). These two sound types were the most commonly heard and therefore selected as the two representative sound types for the study.

Spectrograms of all selected vocalizations were produced using Spectrogram 14 (R. Horn, Visualization Software) with 4096 point fast Fourier transforms (FFT) yielding 5.4-Hz frequency resolution. A series of variables, listed in Table 1, were measured from the spectrographic samples of each vocalization. The variables measured included the vocalization duration, the frequency of the spectral peak (frequency which contained the most energy), and properties of the fundamental frequency. Measurements of the lowest frequency component of the vocalization (the fundamental in harmonic sounds) were: start and end frequencies, minimum and maximum frequencies, ratio of start to end frequency (frequency trend ratio), and ratio of the maximum to minimum frequency (frequency range ratio; see Dunlop et al. 2007 for further details). Ratios of frequencies were measured rather than the differences since ratios better match mammal perception of frequency differences (Richardson et al. 1995). All frequency measurements were initially made on a linear scale and then converted to a logarithmic scale for analysis as these match the perception of pitch in mammals (Richardson et al. 1995). The number of each sound type was counted for the duration the group was audible. Recordings were divided into 5-min time bins and the number of each call type counted within each time bin to give a total number of “wops” and “grumbles” per 5 min. The number of animals (non-singing adults, calfs, and singing adults) was also noted for each time bin.

**Table 1:** Measurements, a description of each measurement, and a summary of the raw values for each parameter (mean and range) for “grumbles” and “wops”

Measurement	Description	Grumble parameters (*n* = 173)	Wop parameters (*n* = 160)
Duration (s)	Vocalization length	1.6 (0.1–9.5)	0.8 (0.4–1.6)
Minimum frequency (Hz)	Minimum frequency	43 (<30–112)	50 (<30–166)
Maximum frequency (Hz)	Maximum frequency	50 (30–132)	78 (37–195)
Start frequency (Hz)	Start frequency	48 (<30–112)	57 (<30–186)
End frequency (Hz)	End frequency	48 (<30–112)	77 (37–195)
Peak frequency (Hz)	Frequency of the spectral peak	48 (30–151)	71 (37–166)
Frequency range (as ratio)	Max freq/min freq	0.2 (0.9–2.5)	1.4 (1.0–3.2)
Frequency trend (as ratio)	Start freq/end freq	1.1 (0.5–3.1)	0.9 (0.4–1.9)
Source level (dB *re* 1 μPa at 1 m)	Vocal amplitude	158 (128–183)	162 (126–183)

All frequency variables were measured on lowest frequency component *F*_0_, apart from the peak frequency

### Estimation of vocal level

The acoustic system was calibrated in situ by inserting white noise and tones of various known levels into the system at the output of the hydrophones and recording this on the hard disk. Hydrophones and the complete systems were also calibrated at the Woronora hydrophone calibration facility near Sydney. This allowed measured recorded data to be converted to received levels at the array in decibels *re* 1 μPa. The (noise-corrected) received sound pressure level (RL) of each humpback whale social vocalization was measured in one third octave frequency bands in the range of 40 Hz–2 kHz, within which almost all of the energy of the vocal sounds was confined. Broadband levels were calculated by summing the mean square voltages in the one third octave bands and then converting to decibels (by calculating 10 times log of the sum). The noise level (NL) in the study area (measured as a broadband level over 40 Hz–2 kHz) ranged from 89 to 105 dB *re* 1 μPa depending on the wind speed (5–20 kn). Passing recreational vessels temporarily (20–30 min) increased the background noise depending on how far offshore they were, though were rarely present when these recordings were made. The median noise levels for this study site was 95 dB *re* 1 μPa, where the wind speed was 12–15 kn and there were no audible passing vessels in the area. Source level (SL) is defined as the estimated radiated sound pressure level at a distance of 1 m from the source and expressed as decibels *re* 1 μPa at 1 m (Table 1). SLs were determined from the RLs by

$$ \operatorname{SL} = {\operatorname{RL}}_{+ } \operatorname {TL}(r) $$

where TL is the transmission loss as a function of distance (*r*) between the vocalizing group and the hydrophone in meters (calculated from each theodolite fix). The transmission loss was determined from a series of measurements of the loss using boats and a J11 underwater acoustic projector as sources (see Dunlop et al. 2013 for details of the transmission loss model). The transmission loss model also allowed the received level of vocal sounds to be predicted at any location in the study site depending on the distance from the source (vocalizing whale) to the location (e.g., singing whale). Received signal-to-noise ratios (SNRs) of the group’s vocal sounds were then calculated at the closest singer to the group by

$$ \operatorname{SNR} = \operatorname{predicted}\kern0.5em \mathrm{R}\mathrm{L}\ \hbox{--}\ \mathrm{N}\mathrm{L} $$

### The composition and behavior of vocalizing groups

A group of humpback whales displayed coordinated surfacing activity and maintained individual separations of no more than 100 m from each other. Humpback whale groups (subject groups) in which “grumble” and/or “wop” sounds were heard were selected for analysis. These were first categorized according to their social behavior based on the land-based observations (observers would have been “blind” to the selection of these subject groups; Table 2) as follows. Subject groups were first divided into “non-affiliating” and “affiliating.”

**Table 2:** Number of each vocalizing group (with number of sounds in parentheses) using “grumbles” and/or “wop” in their repertoire as they traversed the study site

	Grumbles (42 groups)	Wops (34 groups)
Group social behavior		
Single-adult non-interacting groups	7 (25)	9 (28)
Multiple-adult non-interacting groups	13 (60)	11 (58)
Singer-join	9 (36)	6 (26)
Non-singer-join	13 (52)	8 (48)
Nearest singer		
Joined	9 (36)	6 (26)
Within 2.5 km	8 (34)	6 (30)
Between 2.5 and 5 km	8 (23)	7 (28)
Beyond 5 km	17 (80)	15 (76)
Nearest neighbor		
Within 2.5 km	19 (87)	17 (72)
Between 2.5 and 5 km	6 (25)	6 (22)
Beyond 5 km	17 (61)	11 (66)

These vocalizing groups were categorized into four different social behaviors and then re-categorized according to the distance of the nearest singing whale and nearest non-singing group

- *Non-affiliating*—the subject group did not join with other groups or animals for at least 20 min before (as they came within sight of the visual observation team), during (10–30 min), and at least 20 min after vocal sounds were recorded (they were usually out of sight of the visual observation team after this time).
- *Affiliating*—another animal or group of animals joined the subject group while vocalizations were recorded.

The social structure of non-affiliating subject groups comprised either “single-adult” (known males, adults of unknown sex, and adult females with a calf) or “multiple-adult” groups (where there were at least two adults in the group including “adult pairs” and adult females, with a calf being escorted by at least one other animal). Affiliating subject groups either involved a “singer-join” (mostly female–calf pairs being joined by a singing whale) or “non-singer-join” (mostly female–calf pairs being joined by a non-singing [probably] male escort or group of escorts; Table 2). The time of the join was noted as the time when the new animal or animals were sighted within 100 m of the original group. Vocalizations were included in the analysis up to 10 min preceding the sighted join and up to 10 min after the sighted join to incorporate sounds used during the interaction. As singers do not emit social vocalizations while singing, all measured sounds from “singer-join” groups came from the group being joined and not the singer. In “non-singer-join” groups, most of the sounds preceding the join were recorded from the group being joined (rather than the joining animal or animals). However, as these groups converged, it was not possible to separate sounds into those from the group being joined (which usually did not change course) and those from the animals joining the group (who usually changed course to approach the group).

The distances of the nearest singing whale (from the acoustic tracking) and the nearest non-singing whales or groups of whales (from land-based observations) were both measured at the time the vocal sounds were recorded from each subject group. Subject groups were then categorized according to the presence of the nearest singing animal (Table 2).

- *Joined*—groups in which the singer eventually joined; there was no other singer in the area.
- *Within 2.5 km*—the singer came within 2.5 km of the vocalizing group during the time vocal sounds were heard, but never joined. The closest singer was about 1 km from the group.
- *Within 5 km*—the singer came within 5 km from the vocalizing group, but never closer than 2.5 km.
- *Beyond 5 km*—the closest singer was beyond 5 km from the vocalizing group; usually there were no audible singers in the area.

Subject groups were also categorized according to the distance of the nearest non-singing whale or group of whales using the same distance criteria as above (within 2.5 km (the closest being about 1.5 km), 2.5–5 km, beyond 5 km; Table 2).

### Statistical analysis

All analyses were conducted using the statistical software package “R” (R Development Core Team 2012). Measured vocalization parameters (of which frequency measures were logged) and vocal source levels were averaged for each analyzed group to give one independent measure per group. The average number of each call type per group per 5 min was used as a measure of vocal rate. However, as this was likely to be influenced by the number of animals in the group, the average number of each call type per non-singing adult per 5 min was used as a measure of individual vocal rate. It was assumed that calfs and singing whales did not contribute to the vocal rate of these sound types. Therefore, to obtain a measure of individual vocal rate, the number of each call type within each 5-min time bin was divided by the total number of non-singing adults in the group and then averaged over the duration of the recording.

Each group was assigned one social behavior category (single-adult, multiple-adult, singer-join, or non-singer-join), a nearest singer category (joined, within 2. 5 km, 2.5–5 km, and beyond 5 km), and a nearest neighbor category (within 2.5 km, 2.5–5 km, and beyond 5 km). First, to test for differences in vocal parameters (frequency, duration, and source level) with group social behavior, separate linear models (“normal” or “Poisson” based on the distribution of the response variable) were fit. The mean of each vocal parameter per group (response variable) was tested using social structure (single-adult, multiple-adult, singer-join, non-singer-join) as the fixed effect (after checking for normality, unequal variance, and highly influential points and assuming independence of the response variables). Secondly, to test for any “audience effect” on each vocal parameter, the nearest singer and nearest neighbor was separately included as the fixed effect, with each vocal parameter as the dependent. The results of each linear model are presented as *F* values with associated degrees of freedom and adjusted *p* values (using the “p.adjust” function in “R” which adjusts a given set of *p* values using a Bonferroni method). Significance was set at *p* < 0.05 after the correction was applied. Poisson models were compared to the null model to test for significant (*p* < 0.05) improvement using a likelihood ratio test. Test results are presented as *χ*^2^ with associated degrees of freedom and adjusted *p* values. Effect sizes are presented as back-transformed values with 95 % confidence intervals.

Finally, to test whether frequency coding (in “grumbles”) still occurred in the presence of a singing whale, groups were then divided into those in which there was a singing whale within 5 km (16) and those in which there was no singing whale within 5 km (17). Groups being joined by a singing whale were eliminated from this analysis. These groups were also divided into those that were affiliating (groups being joined by non-singing whale(s), 14) and those that were not affiliating (19). Both the presence of a singer and group behavior were included as an interaction effect, with “grumble” maximum frequency and source level as the two dependent variables.

## Results

The acoustic parameters for each sound type are summarized in Table 1. Each group produced between 2 and 12 “grumbles” (with a mean of 5 per group) and between 3 and 21 “wops” (with a mean of 6 per group).

### The effect of group social behavior

**Fig. 2:**
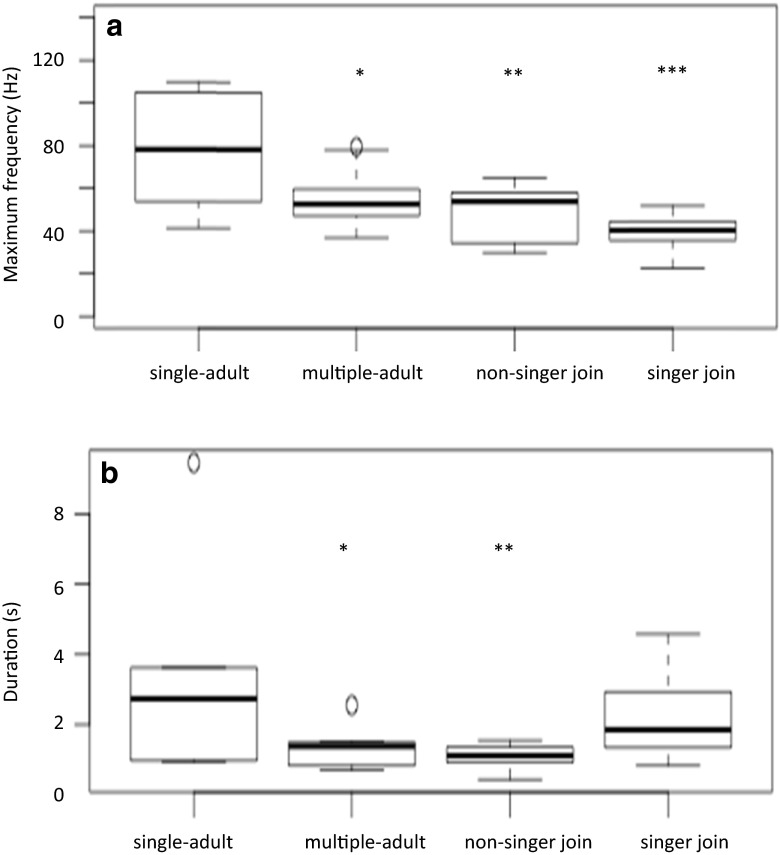
Box plots (displaying the median, lower and upper quartiles, minimum and maximum values, and outliers outside 1.5 times the interquartile range above the upper quartile and below the lower quartile) of the maximum frequency (unlogged) (**a**) and duration (**b**) of “grumbles” recorded from all single-adult non-interacting groups, multiple-adult non-interacting groups, groups involved in a join with a non-singing animal, and groups involved in a join with a singer (with significance set at *p < 0.05, **p < 0.01, and ***p < 0.001 compared to single-adult non-interacting groups)

As “grumbles” were low-frequency, unmodulated sounds (Fig. 1), frequency parameters such as minimum, maximum, start, end frequency, and peak of the fundamental were highly correlated with each other; therefore, only the maximum frequency and peak frequency were analyzed (minimum frequency was excluded as it sometimes corresponded to the noise level and was difficult to measure). The source level and general structure of “grumbles” (the frequency trend and range) were not found to be significantly dependent on group social behavior (Table 3). Neither were the fundamental frequency parameters, including level of “wops” (Table 3). However, groups that were joined by a singing whale (of which the majority were female–calf pairs) vocalized at significantly lower maximum and peak frequencies (Table 3 and Fig. 2a) compared to single-adult non-affiliating groups. “Grumble” maximum and peak frequencies were lower by an average of 39 Hz (95 % CI = 22–56 Hz) and 33 Hz (95 % CI = 13–54 Hz), respectively, in these “singer-join” groups. Groups being joined by a non-singing whale or group of whales also emitted “grumbles” at lower frequencies (by 30 Hz, 95 % CI = 14–46 Hz), as did groups containing multiple adults (by 22 Hz, 95 % CI = 6–39 Hz; Fig. 2a). Groups joined by non-singing whale(s) and multiple-adult groups also emitted significantly shorter “grumbles” (Table 3 and Fig. 2b) compared to single-adult groups. “Grumbles” produced by groups joined by a non-singing whale(s) were shorter by 2 s (95 % CI = 1–4 s); a similar difference was found in multiple-adult groups (of 2 s, 95 % CI = 0.5–3 s).

**Table 3:** Results of linear models (“normal” or “Poisson”) testing the effect of group social behavior, nearest singer, and nearest non-singing group on various vocal parameters of “grumbles” and “wops”

	Sound type	Group social behavior	Nearest singer	Nearest non-singer group
Maximum frequency	Grumble	***F***_**3,36**_ **= 8.82,** ***p*** **= 0.001**	*F*_3,36_ = 2.81, *p* = 0.424	*F*_2,37_ = 1.48, *p* = 1
	Wop	*F*_3,30_ = 0.38, *p* = 1	*F*_3,30_ = 1.07, *p* = 1	*F*_2,31_ = 0.63, *p* = 1
Peak frequency	Grumble	***F***_**3,36**_ **= 5.44,** ***p*** **= 0.024**	*F*_3,36_ = 3.58, *p* = 0.134	*F*_2,37_ = 0.73, *p* = 1
	Wop	*F*_3,30_ = 0.38, *p* = 1	*F*_3,30_ = 1.07, *p* = 1	*F*_2,31_ = 0.63, *p* = 1
Frequency trend	Grumble	*F*_3,36_ = 0.72, *p* = 1	*F*_3,36_ = 0.86, *p* = 1	*F*_2,37_ = 4.66, *p* = 0.176
	Wop	*F*_3,30_ = 1.70, *p* = 0.189	*F*_3,30_ = 0.79, *p* = 1	*F*_2,31_ = 0.72, *p* = 1
Frequency range	Grumble	*F*_3,36_ = 0.84, *p* = 1	*F*_3,36_ = 1.09, *p* = 1	*F*_2,37_ = 3.77, *p* = 0.272
	Wop	*F*_3,30_ = 1.54, *p* = 1	*F*_3,30_ = 1.34, *p* = 1	*F*_2,31_ = 2.55, *p* = 0.752
Duration	Grumble	***F***_**3,36**_ **= 4.66,** ***p*** **= 0.037**	*F*_3,36_ = 1.36, *p* = 1	*F*_2,37_ = 0.90, *p* = 1
	Wop	*F*_3,30_ = 2.18, *p* = 0.936	*F*_3,30_ = 2.04, *p* = 1	*F*_2,31_ = 0.346, *p* = 1
Source level	Grumble	*F*_3,36_ = 0.74, *p* = 1	***F***_**3,36**_ **= 10.21,** ***p*** **= 0.001**	*F*_2,37_ = 0.51, *p* = 1
	Wop	*F*_3,30_ = 0.66, *p* = 1	***F***_**3,37**_ **= 5.68,** ***p*** **= 0.024**	*F*_2,37_ = 1.22, *p* = 1
Call rate (group)	Grumble	***χ*** ^**2**^_**3,42**_ **= 19.60,** ***p*** **= 0.001**	*χ* ^2^_3,42_ = 1.28, *p* = 1	*χ* ^2^_2,42_ = 0.90, *p* = 1
	Wop	*χ* ^2^_3,34_ = 4.06, *p* = 1	*χ* ^2^_3,34_ = 7.31, *p* = 0.504	*χ* ^2^_2,34_ = 3.35, *p* = 1
Call rate (individual)	Grumble	*χ* ^2^_3,42_ = 7.25, *p* = 0.512	*χ* ^2^_3,42_ = 4.64, *p* = 1	*χ* ^2^_2,42_ = 5.67, *p* = 0.464
	Wop	*χ* ^2^_3,34_ = 2.02, *p* = 1	*χ* ^2^_3,34_ = 2.97, *p* = 1	*χ* ^2^_2,34_ = 3.82, *p* = 0.148

The *p* values were adjusted using a Bonferroni correction. Significant results are highlighted in bold

Group “wop” rate (per 5 min) and individual “wop” rate (per 5 min) did not significantly change with group social behavior (Table 3). Group “grumble” rate, however, was significantly (Table 3) greater in multiple-adult groups and groups being joined by a non-singing whale. As individual “grumble” rate was not found to be significantly different (Table 3), the increase in group “grumble” rate was likely due to the increase in the number of vocalizing animals rather than an increase in individual “grumble” rate.

### The effect of nearest singer and nearest non-singing group

**Fig. 3:**
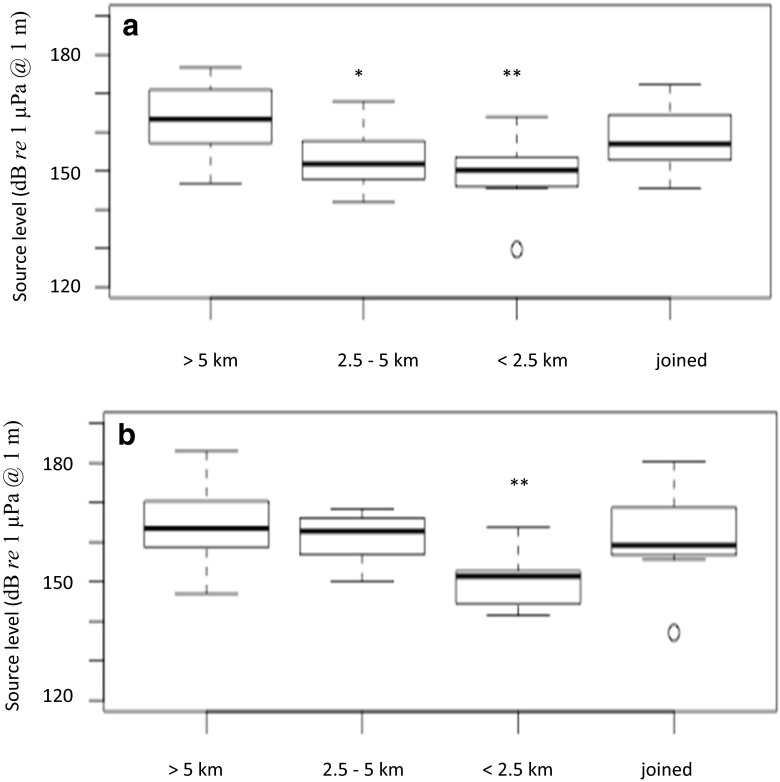
Box plots (displaying the median, lower and upper quartiles, minimum and maximum values, and outliers outside 1.5 times the interquartile range above the upper quartile and below the lower quartile) of the source level of “grumbles” (**a**) and “wops” (**b**) recorded from groups in which the closest singer was within 2.5 km, between 2.5 and 5 km, and beyond 5 km and had joined the group (with significance set at *p < 0.05, **p < 0.01, and ***p < 0.001 compared to groups in which the closest singer was beyond 5 km)

The distance of the nearest singing whale and the distance of the nearest group had no significant effect on “grumble” (or “wop” frequency and duration parameters as well as group and individual vocal rates; Table 3). However, both “grumble” and “wop” source levels were significantly lower (by 14 dB, 95 % CI = 5–22 dB) in groups which had a singing whale within 2.5 km compared to when there was no singer within 5 km (Table 3 and Fig. 3a, b). Groups also emitted “grumbles” at lower levels (by 9 dB, 95 % CI = 2–17 dB) when there was a singing whale between 2.5 and 5 km compared to when there was no singer within 5 km (Fig. 3a). The distance of the nearest non-singing group, however, had no significant effect on “grumble” or “wop” source levels (Table 3).

**Fig. 4:**
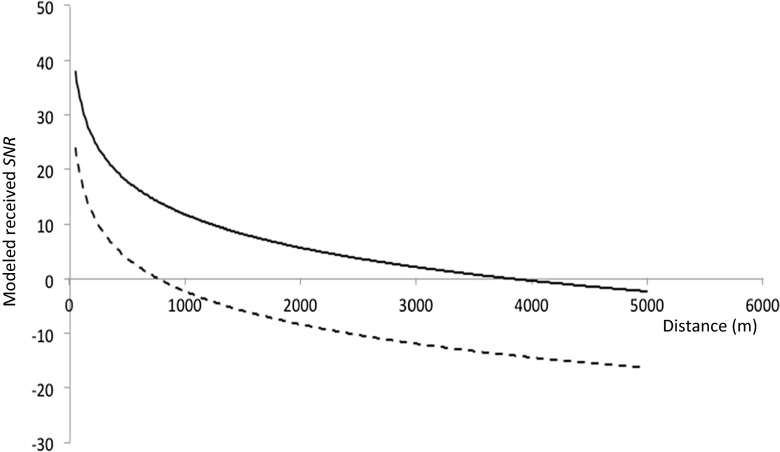
Modeled received SNR of “grumbles” at various distances from the source (vocalizing whale) assuming a SL of 163 dB *re* 1 μPa at 1 m (*solid line*) and a SL of 149 dB *re* 1 μPa at 1 m (*broken line*), with a median noise level of 95 dB *re* 1 μPa

Groups in which there was a singing whale beyond 5 km vocalized at about 163 dB *re* 1 μPa at 1 m compared to only 149 dB *re* 1 μPa at 1 m in groups where there was a singing whale close by. Using the transmission loss model, the received SNRs at various distances from the source were predicted assuming a group SL of 163 dB *re* 1 μPa at 1 m, and again assuming a group SL of 149 dB *re* 1 μPa at 1 m, with a median noise level of 95 dB *re* 1 μPa. At a distance of 2.5 km, the received SNR of “grumbles” was −10 dB in median noise (with a SL of 149 dB *re* 1 μPa at 1 m) compared to 5 dB with a SL of 163 dB *re* 1 μPa at 1 m. This reduction in SL is therefore likely to reduce the ability of a singing whale to audibly detect a vocalizing group at this distance (Fig. 4). The SLs of “wops” was 164 dB *re* 1 μPa at 1 m when the nearest singer was beyond 5 km, decreasing to 150 dB *re* 1 μPa at 1 m with a close-by singer. Therefore, received SNRs of “wops” would also have been close to −10 dB at this distance in the presence of a close-by singer.

### The effect of singer presence on “grumble” parameters in affiliating groups

**Fig. 5:**
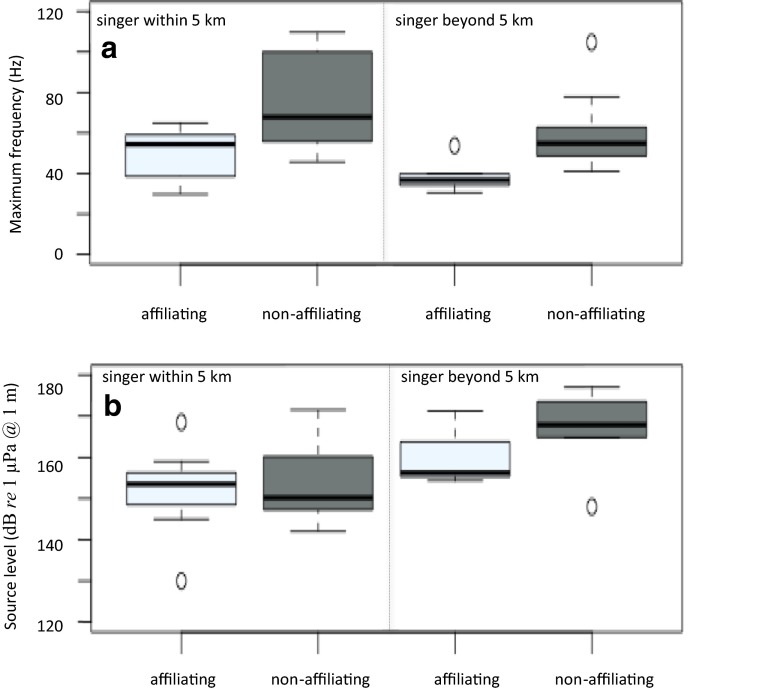
Box plots (displaying the median, lower and upper quartiles, and minimum and maximum values, and outliers outside 1.5 times the interquartile range above the upper quartile and below the lower quartile) of the maximum frequency (unlogged) (**a**) and source level (**b**) of “grumbles” recorded in affiliating and non-affiliating groups where the closest singer was within and beyond 5 km

Affiliating groups, whether or not a singer was present, vocalized at significantly (*F*_3,27_ = 4.24, *p* = 0.014) lower maximum frequencies (by 25 Hz, 95 % CI = 2–47 Hz) compared to non-affiliating groups, suggesting that affiliating groups still emitted “grumbles” at a lower frequency even in the presence of a singing whale (Fig. 5a). Although “grumble” source levels were significantly lower (*F*_3,27_ = 3.58, *p* = 0.026) in groups when a singer was within 5 km (Fig. 5b), source levels were not significantly different between affiliating and non-affiliating groups, even in the presence of a singing whale (*p* = 0.600).

## Discussion

The results show that context-specific differences in vocal parameters may exist in humpback whales and that these differences were related to the sound type, the group social behavior (whether or not they were involved in an affiliation), and the presence of a nearby singing whale. The frequency and duration parameters of “wops” were static in that they did not vary depending on group social behavior. In addition, the general structure of “grumbles” (denoted by the frequency range and frequency trend) was also found to be static. However, the frequency and duration of “grumbles” were found to be flexible and related to the social context of the vocalizing group, implying that these particular vocal sounds fit the frequency code hypothesis (as defined by Ohala 1984). Groups that were involved in an affiliation emitted “grumbles” at lower frequencies and, when comprising more than one adult, emitted “grumbles” of shorter duration. Although this difference in frequency was still evident in the presence of a nearby singing whale (an audible bystander), both sound types were emitted at much (14 dB) lower amplitudes (but at similar rates). This change in vocal amplitude likely resulted in the singing whale being unable, or less likely, to detect the group (since the received SNR of the group’s vocalizations would have been −10 dB at the singer) and could be a mechanism to reduce social eavesdropping by the singer. Nearby groups of whales that were not singing had no effect on group vocal level. Therefore, in humpback whale social communication, the presence of an audible bystander (Marler et al. 1986), rather than a non-singing group, has a significant effect on group vocal behavior in terms of the level at which the signal is produced.

This reduction in vocal amplitude suggests that these groups are acoustically “avoiding” a known (audible) male in the area, an example of vocal crypsis. Vocal rate did not change in the presence of a singer, implying that signals were still being emitted at the same rate, but at lower levels. Therefore, in this “triadic setting” (Zuberbühler 2008), it seems that the bystander (the singing whale) was an untargeted receiver (a bystander). In this study, all recorded groups in which there was a singer close by were comprised a lactating female that would have just given birth to a calf, plus one or more adults. The social structure of these groups would have been either a single male “escorting” the female or groups of males joining and competing for the position of primary escort to the one female (Tyack and Whitehead 1983; Baker and Herman 1984; Brown and Corkeron 1995; Clapham et al. 1992; Clapham 1996, 2000). Since female–calf groups are known to commonly use “wops” and (less commonly) “grumbles” (Dunlop et al. 2008), and both sound types have been recorded from acoustically tagged females (unpublished data from this study site), it is likely, in this study, that the female within the group produced at least some of the measured sounds. One reason for the observed vocal crypsis could be that females are avoiding unwanted attention from singing males, which could be for a number of reasons. Although postpartum estrous in female humpbacks is possible, it is not common (Chittleborough 1958), so these lactating females may not be in estrous and therefore would be unreceptive to males. In addition, recent studies have hypothesized that females with a calf incur energetic costs when being escorted by multiple males (Cartwright and Sullivan 2009; Craig et al. 2014). Further, during aggressive interactions (such as those that occur when females are escorted by a number of males), calves may be injured or separated from their mothers (Baker and Herman 1984; Smultea 1994), indicating another potential cost of joining males. On Hawaiian breeding grounds, female–calf pairs are more likely (compared to females without calves) to be “chased” or “harassed” by males and have been found to avoid playback of sounds from competitive groups (Jones 2010). There is therefore a growing body of evidence that lactating female humpback whales are attempting to avoid male harassment. Lowering the amplitude, but not the rate, of their vocal sounds in the presence of a known male may be one way to do so. There is little research on the call flexibility of females in the presence of bystander males (Townsend and Zuberbühler 2009), though female chimpanzees are known to strategically change their calls according to the social ranking of the female audience (Townsend et al. 2008). Further, in many communication network studies, the audience is also a targeted receiver (e.g., Slocombe and Zuberbühler 2007; Fedurek et al. 2013). The social setting highlighted in this study would provide opportunities to specifically test the effects of an unintended male audience on female vocal activity, such as calls between her and her calf (the intended receiver).

Within the vocalizing group, however, males may also have been producing these sounds (as the acoustic tracking in this study was not accurate enough to localize sounds to a specific group member). The calling rate of “grumbles” was greater in larger groups (which contained one female but greater numbers of non-singing adult males), implying that adult males also emitted these sound types. In terrestrial systems, providing cues to other males in the area can be costly as it encourages competition and can result in reduced mating success (Balsby and Dabelsteen 2005; Stoltz and Andrade 2010). In other words, males within the group may also be acoustically avoiding a known male in the area. Male–female signaling behavior has been found to change in the presence of an audience in various species of fish (e.g., Doutrelant et al. 2001; Makowicz et al. 2010; Dzieweczynsk et al. 2011; Auld et al. 2015), birds (e.g., Vignal et al. 2004), and primates (e.g., Overduin-De Vries et al. 2012; Roberts and Roberts 2015). Hence, in humpback whales, the reduction in vocal amplitude (be it from the female or males in the group) may be to discourage more males (singing whales) from joining, resulting in fewer aggressive interactions between competing males and less within-group competition between males. Interestingly, there was no such effect with the presence of a nearby non-singing whale or group of whales (which may not have been detectable to the group if not vocalizing). The reduction in vocal amplitude found in this study seems to only occur in the presence of bystanders that are audible to the group, i.e., singing bystanders. Whether or not the nearest non-singing groups were audible to the study groups (i.e., producing audible social sounds) was an unknown factor in the study. Further studies should therefore determine whether there is in fact a “cost” to group members of additional males joining and if this “cost” is reduced by acoustically avoiding known (singing) males in the area.

The dataset in this study also included vocalizing female–calf pairs that had already been joined by a singing male, suggesting that not all females were avoiding singing whales or that their avoidance strategy was not always successful. In these groups, the analyzed sounds would have come from the female or calf (singers do not emit social sounds while singing). Given that calves tend to make short-duration (<0.5 s) sounds that are either pulsed, amplitude-modulated, or, if frequency-modulated, tend to be above 500 Hz (Zoidis et al. 2008), it is likely that most of the sounds were from the female in the group. Interestingly, these escorted female–calf groups vocalized at similar levels to groups in which there was no singing whale in the area, illustrating that the observed lowering in vocal amplitude was in response to a close-by singer (that did not become part of the group) rather than to a singer which was already part of the group. Associations between males and females at this study site are common, probably due to males prospecting for mating opportunities (Mobley and Herman 1985; Clapham 1996), implying that at least some of the females would be receptive some of the time. Currently, it is not possible in humpback whales to determine if the physiological state of the female (i.e. whether or not she is in estrous) plays a role in the different vocal behaviors observed, but relating physiological state to vocal behavior may help to further understand the observed variation in vocal amplitude with social context.

In addition, this study has provided evidence that one of the most common vocal signals of humpback whales, “grumbles,” has a static–dynamic component and therefore is likely to contain flexible information such as motivation. “Wops” were not found to be structurally flexible, at least during group affiliations, indicating that these sounds may have evolved to encode specific static information related to their function. In terrestrial systems, low-frequency unmodulated sounds often code for signaler size (Fitch 1997; Vannoni and McElligott 2008; Taylor and Reby 2010) and harsh low-frequency sounds signify aggression (Morton 1977; Reichert and Gerhardt 2013; Ordoniez-Gomez et al. 2015). Low-frequency “rumbles” in elephants are used for contact calling, herd assembly, social interactions, and aggressive interactions (Nair et al. 2009), as well as advertising individual identity, reproductive state, emotional state (Soltis 2010), and social role within a group (Soltis et al. 2009). In humpback whales, “grumbles” are more commonly heard in the multiple-adult groups described above, many of which contain competitive males (Dunlop et al. 2008). “Wops,” on the other hand, are more common in female–calf pairs and female–calf pairs being escorted by a male (Dunlop et al. 2008) compared to other group compositions. In keeping with terrestrial communication systems, perhaps the lower-frequency “grumbles” signify levels of aggression or indicate differing social roles within the group, whereas “wops” reflect a static signaler trait such as sex and/or location. In the Hawaiian breeding grounds, female–calf pairs can be subjected to aggressive advances from males (defined as “herding,” “chasing,” and “blocking”; Jones 2010), suggesting that inter-sex conflicts, as well as intra-sex conflicts (between competitive males), are a common occurrence in humpback whale breeding behavior. At this stage, the function, or functions, of “grumbles” and “wops” in humpback whales remains speculative, as does the reason for the differences (or lack of differences) in frequency during social affiliations. What is clear from this study is that, in certain social situations in humpback whales, it may not just be the type of sound being used that provides the important information, but also the frequency and/or amplitude at which it is produced.

The results of this study highlight the complex communication networks in marine mammals (Janik 2005). Specifically, this study has shown that within the communication system of humpback whales, socially driven dynamic vocal frequency coding exists. This coding system is similar in some ways to a typical terrestrial system in that some features, such as frequency, change with social context, while other structural features do not. Few studies have determined the effects of an untargeted bystander on signaler behavior (Zuberbühler 2008). In humpback whales, it seems that changes in frequency between affiliating and non-affiliating groups still occurred in the presence of an audible bystander; in other words, the coding system remained. However, although the presence of this bystander had no effect on the rate of production of these vocal sounds, sounds were emitted at substantially lower acoustic levels. This reduced signal level implies that humpback whales are suppressing their vocalizations to acoustically “avoid” nearby males. The function of these sounds, as well as the reasons behind the observed differences in vocal parameter, remains, at this stage, speculative and the hypotheses laid out in this discussion are by no means exhaustive. However, the results provide a basis to carry out more targeted research on the function of these sounds in humpback whales and the potential costs to the signaler of eavesdropping by other conspecifics.
